# Expert Consensus on the Resource Needs of Autistic Children and Young People in Ireland During the Early Months of the COVID-19 Pandemic

**DOI:** 10.1007/s10882-023-09904-w

**Published:** 2023-03-24

**Authors:** Cillian Egan, Nadine McLaughlin, Maria McGarrell, Cathal Gurrin, Sarah Devlin, Sinéad Smyth

**Affiliations:** 1 School of Psychology, Dublin City University, Glasnevin, Dublin, Ireland; 2 St Ultan’s, Primary School Cherry Orchard, Dublin, Ireland; 3 School of Computing, Dublin City University, Dublin, Ireland; 4 National Council for Special Education, Dublin, Ireland

**Keywords:** ASD, Autism Spectrum Disorder, COVID-19, Transition, Resources, Consensus, Collaborative research

## Abstract

The COVID-19 pandemic has brought significant changes across society. This Delphi study aimed to gain expert consensus on challenges faced and resource needs for autistic children during the COVID-19 pandemic. Round 1 of the Delphi method employed semi-structured interviews with experts (N = 24) which were thematically analysed in order to identify needs, resource targets and resource development. In a follow-up Round 2 survey participants rated emergent need and resource in order of priority. Round 2 provided consensus on challenges faced with anxiety, routine and wellbeing ranked most important. Direction on resource design was also obtained. Consensus on the challenges and resources was achieved and is being integrated into a needs-based transition resources toolkit. Future studies could make use of the Delphi method to quickly gain consensus on focus of needs in other contexts and communities.

## Introduction

The impact of COVID-19 has been far reaching. From the direct impacts on people diagnosed with it and their loved ones, strain on health care systems to more indirect impacts on the lives of individuals worldwide. The true extent of these are difficult to measure may not truly be known for many years to come, if at all. Research has demonstrated that autistic individuals have been substantially affected by the pandemic and associated restrictions (Colizzi et al., 2020). In particular autistic children and young people have been affected by the closure of schools and move to remote learning. It stands to reason that additional resources and supports are needed at this time and while Individuals and agencies have quickly worked to develop these, the ongoing nature of restrictions meant that a few months into the pandemic it became apparent that a more nuanced assessment of needs was required.

### COVID-19 Specific Resources

As the COVID-19 pandemic is quite a recent event, research focusing on this point in time is just now beginning to grow. Autism related COVID-19 research has taken a number of different approaches. Some studies focused on parental reports (Colizzi et al., 2020; Stankovic et al., 2021; Tokatly et al., 2021), while many others were limited to small sample sizes or case studies (e.g., Sakamoto et al., 2021). Yet others focus on recommendations rather than empirical research (e.g., Bellomo et al., 2020; Tarbox et al., 2020). Some research has addressed resource, or service, provision however such studies mainly focused on telehealth interventions (e.g., Narzisi, 2020; Sivaraman, Virues-Ortega & Roeyers, 2021) including interventions mediated by parents (Bhat et al., 2021; Sengupta et al., 2021, Srinivasa et al., 2021) These resource models have been broadly successful and may be of great importance during the pandemic however, it is noted that they should not replace in-person interactions and may not be suitable in certain conditions. There is a need for further research on the effectiveness of non-telehealth interventions and resources for autistic children and their families. It is also important to collaborate with these users and families in resource development, and not just implementation, in order for them to be as effective and useful as possible for as many people as possible.

### Collaboration in Resource Development

To date, there is no known research which has focused on needs based development of resources for autistic children and there is significant need for such research and resources (Mutluer et al., 2020). Despite resources being made available online by a number of organisations working in the area, there is little evidence base for the direction of the development of these resources. The current study aimed to address this issue. Collaborative user design is a development that has been beneficial for many populations. Autistic people are often not part of the design process for technology created for them, although efforts in this regard are increasing (Pellicano et al., 2018). This often leads to inadequate finished products (Millen et al., 2010). With increasing recognition of neurodiversity and consideration of autism as a condition rather than a disorder, it is even more obvious that this is considered an integral part of the research process. Input from the target group for the purposes of this study involves the voice of the child through that of the caregiver and the professionals working in the area. Many of the resources designed for autistic children are used under the supervision of these adults or directed by them. It is important to include a broad range of these individuals. This group may consist of parents, siblings, teachers, psychologists, SLTs, OTs and other professionals.

Collaborative design should be carefully distinguished from post hoc appraisals of experiences of using resources. Such analyses are useful in assessing existing resources but good practice in new resource development includes stakeholders in all stages (Rabba et al., 2020) and as such, assessing resource needs is an important step (O’Neill et al., 2020; Rabba et al., 2020) conducted research examining the impact of collaborative or participatory design in the creation of online resources for parents of newly diagnosed children with autism. This involved a four stage approach, (1) cooperative researcher-stakeholder planning, (2) cooperative researcher-stakeholder–based action, (3) stakeholder observation, and (4) cooperative researcher-stakeholder reflection. The researchers were then able to develop prototypes of the online resource in conjunction with the participants. Participants in the study were made up of parents; researchers and clinicians. Six key topics arose during the study; understanding autism, accessing services, support, gaining funding, combining it all, and future progress. This demonstrates the importance of collaboration in all stages of resource design. Furthermore, reflecting the diversity of autistic children is more likely when stakeholder voicers are included and this was one key goal of the current research.

### The Current Research

The aim of the current study was to assess the challenges and needs of autistic children and their families during the Covid-19 pandemic. This study also sought consensus on the development of resources to address these challenges. Specifically, consensus was sought on the greatest needs and targets, and prioritisation of resource development to combat these challenges. We set out to achieve this using the Delphi Method. The Delphi Method is an iterative process, seeking the views of experts in the area (Cantwell et al., 2018). A number of characteristics define a Delphi study. A panel of appropriate experts must be recruited. Delphi studies are undertaken over a number of rounds and earlier rounds should help to inform the design of subsequent rounds. In these later rounds the panel will be able to rank the findings of previous rounds in order to form a level of consensus. At the time of writing, this is the first study on the challenges faced by autistic children during COVID-19 also looking at the identification of resource needs. The inclusion of experts by experience as well as experts through practice and training offers unique insights into the situation.

## Method

### Design

The current study forms part of a larger research project examining the experiences and needs of autistic children and young people through the early years of the pandemic (blinded: authors, 2021). This particular phase focused on determination of supports needed. A traditional Delphi method was used in this study to achieve consensus on the challenges in returning to school and dealing with the changing restrictions due to COVID-19 for autistic young people and their families. Ethical approval for this study was granted by the Dublin City University Research Ethics Committee.

### Participants

Participant numbers in Delphi studies reported in the literature vary widely (Vernon, 2009). Despite this wide discrepancy, the majority of papers appear to recruit within a tighter range of between eight and thirty-four participants (Trevelyan & Robinson, 2015; Okoli & Pawlowski, 2004; Czinkota & Ronkainen, 1997; Kuo, 1999). Group dynamics for achieving consensus rather than statistical power, tend to drive group size in delphi studies (Okoli & Pawlowski, 2004). Furthermore, representativeness in the Delphi method is based on the qualities of the panel of experts and not the number of experts involved. As such the participant group does not need to be representative in the statistical sense (Powell, 2003). Twenty-four participants (22 = female, 2 = male; see Table 1) were recruited for Round 1. All were adults aged 18 or over. Two of the participants in the Delphi study identified as autistic. Four participants were parents of autistic children. The remaining 18 participants were professionals working in relevant areas. These roles included occupational therapists, speech and language therapists, psychologists, teachers and other experts. Twenty-one responses were obtained for round 2 due to attrition of three participants (see Table 2 for an outline of the participant roles). Purposive recruitment was undertaken through emails to schools and organisations that work with autistic children and their families. Promotions were circulated on social media and the study website (Autism-Toolkit.ie) and finally snowballing was employed to increase participant numbers.

**Table 1:** Participant Demographics

Variable	No. (%)
Gender	
Male	2 (8.3%)
Female	22 (91.7%)
**Age**	
20–29	9 (37.5%)
30–39	8 (33.3%)
40–54	7 (29.2%)

**Table 2:** Participant Roles & Experience

Variable	No.	Mean years of experience (Range) SD
Role		
Expert by experience	6	-
Teacher	4	10.5 ( 8–15) SD = 3.11
Behaviour specialist	3	6.67 (1–13) SD = 6.03
Psychologist*	2	20.50 (16–25) SD = 6.36
Occupational Therapist (OT)	5	7.20 (1–19) SD = 7.22
Speech and Language Therapist (SLT)	4	10.0 (1–21) SD = 8.29

* one participant was both a sibling of an autistic person and a Psychologist working with the population. They were counted as a psychologist, however, their experience in years was given in terms of years as a sibling

### Materials

Round 1 interviews were conducted online via Zoom. The interview schedule consisted of both open and close ended questions which participants were asked to respond to with autistic children aged 4 to 18 years old in mind. The first 11 questions collected basic demographics and background information. The remaining questions explored difficulties faced by children and their families due to COVID-19 and associated restrictions, resources and strategies to alleviate difficulties, and optimum means of dissemination and promoting uptake (See appendices for full list of questions). The Round 2 survey sought to achieve consensus from participants on the challenges faced and resources needed. This was conducted via an online survey emailed to Round 1 participants. The survey consisted of eight questions.

### Procedure

In-depth semi-structured interviews were conducted with participants through online video calls between July and August 2020. By this time, children had been out of school for at least three to four months. These interviews were recorded and then transcribed by a third party service. Each participant was interviewed individually except for two co-parents who were interviewed together. At the end of the interview, participants were given the opportunity to identify any further challenges, resources and/or topics of exploration that were not previously addressed in the interview questions. Interviews were conducted and recorded using Zoom software and applications.

The results of a thematic analysis of the Round 1 interviews provided the basis for a Round 2 survey conducted in October 2020. In Round 2 respondents were provided with a link to the online survey. First they were present with a summary of the results from Round 1 and were then asked to complete all questions. Participants were first asked to rank seven predicted challenges identified in Round 1 in order of importance. They were also asked to rank six specific potential targets of resources, six modes of resource presentation and six desirable features of resources. Next, participants were asked to rank methods of promoting uptake of such resources. Participants were asked to consider their own opinions and experiences, when ranking the variables. There were also three open ended questions for participants to raise any issues they felt had not been adequately addressed. Qualtrics software was used to conduct the questionnaire.

### Community Involvement

This study forms part of a larger project which was designed in collaboration with an autism advocacy charity as well as practitioners. This actual study involved integrated knowledge translation with knowledge users (in this case educators) involved in all aspects and listed as co-authors.

## Results

### Delphi Round 1 Analysis

#### Challenges Identified

An inductive thematic analysis was conducted and the themes which emerged were Changing routines, COVID-19 specific issues and Behavioural concerns. A content analysis provided the priority behaviours and resources which were the basis for resources developed as part of the wider project.

### Changing Routines

Of concern for many respondents was the theme of changing routines. The first subtheme under this was the return to school and the community after a period of restricted activity. Participants spoke with reference to a long period of remote learning (March to end June 2020) as well as a period of restrictions during which household visits were curtailed, non-essential retail, sports and personal care services (e.g. barbers) were closed and public transport was reduced to 25% capacity. Seventeen of the 24 participants in the study specifically highlighted the transition back to school in particular as one of their main concerns *“And a lot of the anxieties school brings, such as the social demands and the academic demands, are going to return now. So that would be a big challenge”* (Participant 4, OT and Autism Training Advisor). *“I think that we’re expecting to see a huge amount of school refusal which we’re trying to prepare for…”* (Participant 2, psychologist in clinical training). Being prepared for future change and transition extended beyond the return to school. How to deal with future restrictions should they be implemented within the community was another concern, *“obviously the possibility of another lockdown and again we’re going to go back into that like you know massive change to routine with no notice is stressful so I suppose like being able to prepare them as much as you can for the possibility of another lockdown” (*Participant 11, OT). Routine changes also concerned many of the services outside of school with which the children and young people would normally engage and this was considered a second subtheme under routine changes. Initially, these were postponed due to COVID-19 restrictions and uncertainty predominated as to whether these services would resume and in what format This theme of access to services was highlighted in four interviews. *“families having access to supports at the moment is still very unknown*” (Participant 3, OT).

### COVID19 Specific

Since the arrival of COVID-19 to Europe in 2020, rules and guidelines implemented have significantly changed life for the public. Social interactions are altered with social distancing, mask wearing, limiting the numbers of contacts and no physical contact amongst people. The continued changes to guidance as the government moved between levels made adjustment difficult. This issue was reported by 17 participants and subthemes identified within the theme of adjusting to COVID19 rules and guidelines were; understanding and adherence and anxiety/fear.

The first subtheme, issues around understanding and adhering to the guidelines were highlighted by 16 participants. Social distancing, mask wearing, sanitisation and restricted travel had all become part of life in Ireland. However, public health guidelines changed frequently since the onset of the pandemic and communication was not always clear. Many of these were complex concepts and behaviours which represent a big change in the way people conducted themselves. A significant change from previous ways of life. Understanding of the changes as well as learning to adhere to them were a serious concern. “*But now that it’s kind of unclear he is kind of reverting back to going closer to people and I would like other people to engage as well and not automatically go oh, maybe I should just let it go because he’s Autistic…. oh you know, we’re allowed go to X, Y and Z now and whatever else and I keep reinforcing that at the moment, yes we can maybe another day we’re not going to be able to do that.”* (Participant 12, Parent of autistic child).

Participants highlighted that difficulties engaging with the guidelines could be disabling in indirect ways. For example, being unable to follow the guidelines could place an obstacle in access to other activities or services. For example; *“she won’t wear a face covering and that’s disabling for her because it means that she can’t access places like shops or public transport…*” (Participant 2, Psychologist in clinical training with an autistic family member). Despite allowance for certain groups, it could still be difficult to access premises or transport if there were issues adhering to social distancing, personal protective equipment or other regulations. Some have had problems with sensitivity around hand sanitizer and mask wearing which became important aspects of the guidance in preventing infection. Three of the interviewees spoke about sensory problems. “*The sensation of the mask on the face like, she’d be quite sensitive with her clothes and things like that you know”* (Participant 2).

It was reported that rule breaking by others was an issue for some children. *“.People breaking the rules around social distancing can be quite upsetting to them.”* (Participant 7, SLT). People failing to follow guidelines could increase anxiety around safety for some students while for others there was confusion as to why someone would not follow the rules. …” *expecting everybody you know if you wear a mask everybody has to wear a mask in every context. So, having huge difficulty understanding… I suppose the different way individuals interpret the restrictions*” (Participant 13, Educational Psychologist). Rule breaking with regard to restrictions was not uncommon throughout the pandemic.

General fears that students had around safety in the pandemic and of the virus were mentioned in the second subtheme. Participant 8 (SLT) spoke of *“the fear over COVID as well and germs.”* There was also anxiety in relation to the uncertainty caused. *“The uncertainly of all of it will definitely be a challenge to those that are diagnosed with ASD [Autism Spectrum Disorder]*” (Participant 6, OT). Similarly, anxiety levels were a concern within families in relation to the virus and the pandemic and more specifically around getting sick. One participant spoke of the general anxiety. “*So, I would imagine the pandemic itself has probably created quite a bit of anxiety for families and for young people with autism*” (Participant 9, SLT). While another spoke of the fear of sickness not only for the child themselves but also for their family members. “…*there’s a huge focus I suppose on will I get sick or will my relatives get sick*” (Participant 3, OT).

### Skills Decline

There was significant concern amongst participants surrounding stagnation or regression in skills and abilities for their children brought on by the pandemic and associated restrictions. Concerns were highlighted across three subthemes; social skills, emotional regulation and sensory sensitivity. Nine participants were particularly worried about regression in social skills with a further four concerned about communication skills. “*We saw quite a lot of regression in behaviour and communication and that’s one thing I am quite worried about* (Participant 1, ABA Teacher). “*They saw a bit of regression in kind of daily living skills*” (Participant 7, SLT**). “***I think it would have a massive impact on their ability to make friends and initiate social situations*” (Participant 1). “*One significant concern for the future is that having been confined to home and having very little sensory or social challenges, children may struggle in returning to school and wider involvement in society*” (Participant 4, OT). With the lessening of restrictions, this would change again and they would face more social situations, noise and other sensory stimuli which may prove an obstacle. Two participants expressed concerns about emotion regulation. **“***if there was another lockdown to happen that would be really tough for a lot of students*“ (Participant 8, SLT). It was apparent that the impact of potential future lockdown and the lessening of restrictions were causes for concern.

### Summary of Anticipated Challenges

In summary, three themes emerged; changes to routine, COVID-19 specific concerns and declines in skills and abilities. Each of these was further broken down with school and community routines as well as access to services emerging from the broader theme of changes to routine. COVID-19 specific concerns were broken down into issues with understanding around public health rules and mitigation strategies as well as anxiety and fear about COVID-19. Finally, the theme of skills declines highlighted three subthemes; social skills and communication, sensory sensitivity and emotional regulation.

### Target Behaviours and Resources

Earlier questions in this round focussed on challenges faced. The final section of questions focussed on issues or behaviours that should be the focus of resources that may be created in order to mitigate these challenges. Participants were asked about resources in terms of what they should target, resource mediums, resource features and dissemination media. It is important that resources developed are targeted at the most appropriate areas for children and their families in order for them to be most effective and it was with this aim in mind that this data was analysed. A content analysis (Stemler, 2015) was used in this section to identify the most important targets, resources and dissemination methods. This was deemed the most appropriate means of collating and quantifying the findings of this section which was identifying resource needs rather than an exploration of concerns as in the case of our consideration of the challenges faced.

Dealing with uncertainty was the most common target for resource development with 15 participants highlighting this area “*uncertainty and Autism don’t really go together, you know, they’re a bad mix for those people with Autism…*” (Participant 15, SLT). This was followed by resources for new routines surrounding the COVID-19 restrictions being spoken about by six participants. These kinds of routines included hand washing, mask, social distancing, getting the bus, etc. Other topics raised included Transitioning to a new school setting (3) daily living skills (toileting, self-care etc.) (1), getting a test/ getting sick (1), online learning (2) and wellbeing (2).

In terms of the most appropriate format for resources, videos and webinars were most popular with nine mentions followed by educator supports (7), PDFs (printables) (5), visual supports (4) social stories (3), animations and comic strips (3) and podcasts (1). With regard to resource features, adaptable templates were most popular (8) followed by visuals with text (6), central system (5) short & condensed (4) relevant content (3) ability based (Hierarchy of levels) (3) age appropriate (2).

Means of disseminating the data received quite an even endorsement from participants with social media posts, paper-based materials and previously tested resources all receiving backing from two participants. Dissemination by contacting professionals directly and through school contact were endorsed by one participant each.

### Results Delphi Round 2

Measures of consensus vary and there is no universally agreed definition (Hasson et al., 2000). In the current study, and based on previous literature in the area (Junger et al., 2017; Okoli and Pawlowski, 2004), the criteria for consensus was set with a cut off of over 70% agreement. Specifically, we defined consensus as a response option being ranked in the top three by more than 70% of participants. This was because the aim of the Delphi study was to gain consensus on resource priorities, with a view to focusing on more than one resource.

Participants were first asked to rank seven predicted challenges (anxiety”, “changes to school and regular routines” “understanding COVID-19 rules and guidelines”, “accessing external services”, “social skills and communication”, “emotional regulation” and “ability to manage sensory sensitivity”), which emerged in Round 1, in order of how they should be prioritised for intervention. Anxiety was ranked in the top three most important issues by 75.9% of respondents and interventions targeting “changes to school and regular routines” was also selected by 75.9%. The other options which received lower rankings included “Understanding and understanding COVID-19 rules and guidelines”, “accessing external services”, “social skills and communication”, “emotional regulation” and “ability to manage sensory sensitivity” (see Table 3 for full details).

**Table 3:** Consensus on the ranked prioritisation of challenges faced

Interventions	% rated in top 3 (*n)*
Anxiety	75.9 (16)*
Changes to school and regular routines	75.9 (16)*
Understanding Covid-19 rules and guidelines	9.5 (2)
Accessing external services	28.6 (6)
Social skills and communications	33.3 (7)
Emotional regulation	52.4 (11)
Ability to manage sensory sensitivity	23.8 (5)

* Consensus (over 70% of participants ranking in the top 3) was achieved for these response options

Participants were then asked to rank six (“Understanding if we get sick and need a COVID-19 test”, “Daily living skills”, “Transitioning to new school setting”, “Understanding new routines”, “wellbeing, including physical, emotional and mental” and “understanding and coping with uncertainty”) specific resources in order of priority for their development. The following were highlighted as important to address in any resources developed. 81% highlighted “wellbeing, including physical, emotional and mental”. 75.9% chose “understanding and coping with uncertainty”. This option included coping with further lockdowns and school closures. The other rankings can be seen in Table 4. Of the six options in the third question on types of resources, the most popular types of resources amongst participants were “videos and animations”, which were ranked in the top 3 by 90.5% and “visual supports”, which were ranked by 71.4%. The percentage of participants ranking the other response options in the top three can be seen in Table 5. In the fourth ranking question, participants were asked to rank their preference for different resource features. Short, condensed and user-friendly styles of resources were the only resource feature which was ranked above the consensus cut off point of 70% of participants ranking it in the top three. This option was selected by 71.5% (see Table 6 for % top three rankings for the other response options). The most popular of the four strategies to promote use of resources were “contact from schools and support groups” (90.5%) and “recommendations by other users such as teachers, parents, etc.” (90.5%). The lower ranked options included “Social media posts” and “Coverage in national or local media (radio, newspapers etc.)”. As consensus was clearly achieved following two rounds, there was no requirement for any further rounds.

**Table 4:** Consensus on the ranked prioritisation of specific resources that should be developed

Interventions	% participants who rated in top 3 (*n)*
Understanding new routines (e.g., Hand washing, social distancing, mask wearing)	28.6 (6)*
Transitioning to new school setting (e.g., Primary to secondary, secondary to adult services)	47.7 (10)
Daily living skills (e.g., independent skills such as cooking, washing up, going to the shops etc.)	38.1 (8)
Understanding and coping with uncertainty (e.g., future lockdowns, school closures)	75.9 (15)*
Understanding what happens if we get sick or need a COVID-19 test	14.3 (3)
Wellbeing (including physical, emotional and mental aspects)	81 (17)*

* Consensus (over 70% of participants ranking in the top 3) was achieved for these response options

**Table 5:** Consensus on the ranked preference for resource types of specific resources that should be developed

Interventions	% participants who rated in top 3 (*n)*
Visual Supports (e.g., Visual schedules, calendars)	71.4 (15)*
Videos (e.g., animations for young people)	90.5 (19)*
Social Stories (incl comic strips)	47.7 (10)
Podcasts	4.8 (1)
PDFs (Printables)	28.6 (6)
Webinars (aimed at teachers, parents, professionals)	38.1 (8)

* Consensus (over 70% of participants ranking in the top 3) was achieved for these response options

**Table 6:** Consensus on the ranked preference for resource features

Interventions	% participants who rated in top 3 (*n)*
Irish context specific	23.8 (5)
Ability specific	52.4 (11)
Age specific	33.3 (7)
Visuals accompanying text	57.1 (12)
Templates (resources that are adaptable)	52.4 (11)
Short and condensed	71.5 (15)*

* Consensus (over 70% of participants ranking in the top 3) was achieved for these response options

## Discussion

Disasters and emergency situations tend to affect the more vulnerable members of society disproportionally. To the best of the authors’ knowledge, this study is the first to explore the resource needs of autistic children and young people during the COVID-19 pandemic. Broad challenges as well as targets and design for resources were identified clearly through consensus.

### Identification of Areas of Need

The purpose of this study was to identify and gain expert consensus on areas that are of most immediate concern for autistic children and young people as they navigate the COVID-19 pandemic. A number of key areas emerged and there was clear consensus on these following the two rounds of this Delphi study. Unsurprisingly, school closures and reopenings have been a concern for many students throughout the pandemic. This theme was prevalent in Round 1 of the study, while Round 2 provided consensus of this with the high ranking of both “Changes to school and regular routine” and “Understanding & coping with uncertainty (e.g. Future lockdowns, school closures)”. With the increased levels of reliance on routine for autistic students (Mutluer et al., 2020), these challenges have been amplified by the pandemic. Closing schools significantly changed the day-to-day lives of students. Many developed new sleeping, rising, screen-time and eating routines. There was also a distinct reduction in sensory stimulation and social interaction as people were forced to limit contacts and became increasingly confined to their homes.

The high levels of concern surrounding routine and sleep patterns in the current study align with other research findings in relation to the reaction to the pandemic amongst autistic people, their families, teachers and therapists. The highest priority of resources highlighted were in relation to changes of routine and coping with uncertainty. These findings will be incorporated in resource design arising from the study. Nuske et al. (2019) found that predictable school transitions such as from primary to second level school can be challenging for autistic children. Therefore, it stands to reason then that the sudden and dramatic changes to schooling and general life caused by COVID-19 could cause significant problems. Mutluer et al. (2020) found that autistic children slept less hours at night since the onset of the pandemic in comparison to before. A sense of anxiety emerged amongst participants in this study surrounding the reopening of schools with fears around school refusal, sensory overload in the readjustment and other issues.

Although the issue of navigating restrictions and public health guidelines brought in during the COVID-19 pandemic was highlighted in Round 1, it did not feature in the top ranked issues from Round 2. It is possible that this change in priorities arose from the normalising of the changes over time. Public health measures such as mask wearing, hand hygiene and social distancing have become part of everyday life for most people since their introduction. Despite this, research has found that autistic people can have a different understanding of COVID-19 and the restrictions (Mutluer et al., 2020). This, coupled with the importance of routine has made the adjustment to society with COVID-19 restrictions more difficult. Findings confirm there have also been some difficulties engaging with some of the infection-preventing measures that have been implemented. Examples of this include sensory issues with masks and hand washing which are not surprising given the profile of this population (Kojovic et al., 2019). One interesting point that this raises is that as we continue to progress through the pandemic, different public health concerns may arise and so consultation on experiences and needs should continue. At the time of data collection for example, vaccinations were at test stage and had not been approved for use, thus were not raised as an issue by our participants. It is possible that this or other issues may arise now and later on in the pandemic.

Anxiety was a common theme in Round 1 and ranked highly amongst the priorities of participants in Round 2 of this study. This was not unexpected given the centrality of the issue in related studies. Participants also voiced their desire for resources dealing with physical, emotional and psychological wellbeing. A study of autistic adults in the USA who were capable of providing self-report, compared mental health changes from immediately prior to the declaration of the COVID-19 pandemic and two months later. The research by Adams et al. (2021) showed that almost two thirds of participants had reported some kind of COVID-19 related distress. It also found that higher levels of distress were correlated with increases in anxiety and depression symptoms. Colizzi et al. (2020) reported increased challenges in the population and psychological distress. Behavioural changes were noted with regression reported in areas such as managing daily activities, more intense and more frequent behavioural dysregulation. More significant needs pre-pandemic were associated with increased regression.

Mutluer et al. (2020) found that behavioural issues in autistic children had increased during the pandemic and that this was correlated with increased levels of anxiety amongst caregivers and family members. Parental anxiety was strongly associated with measures of lethargy and social withdrawal for their children. It seems to be impacted by a parent’s sense of regression in their child’s skills and abilities. This is something that has been displayed in the current study with many participants voicing concerns around the regression in children’s abilities, the increased challenges posed in interacting with society with the new restrictions and the reduction or in many cases cessation of supports. Parents of autistic children generally exhibit higher levels of stress than parents of neurotypical children (Silva & Schalock, 2012). However, social support is a significant protective factor against this stress. With the reduction in social interactions due to the pandemic restrictions, the levels of anxiety in families would be expected to have risen. Resources designed to address these issues would be appropriate in interventions to assist children and their families in navigating the pandemic.

In Round 1, many participants spoke of their concern for a drop in skills and abilities amongst autistic students during the pandemic. These issues are explained due to the interruption of intensive behavioural and educational interventions that teach and maintain new skills and coping mechanisms. It is imperative that resources are developed in order to try to combat some of these impacts. The reliance of autistic students on routines such as attending school and coming home at set times, means that the disruption to this can have knock effects for their wellbeing and development (Mutluer et al., 2020).

### Collaborative Resource Design

There is a distinct lack of research on COVID-19 resources for autistic children in the existing literature. This is not surprising given the recency of the issue but critically, the current study will help to address that gap and also sign post the need for future collaboration as we navigate this current time. Mutluer et al. (2020) highlighted the need for targeted, distance special education resources, interventions and other supports in the face of the new COVID-19 related changes in society. Often the stakeholders in resources for the autistic community do not have sufficient input in interventions designed for them Rabba et al. (2020). With this in mind, the voice of users was central to the current research. Not only were the areas of concern identified, specific information on preferences for resource design was also gathered and considered. Furthermore, views from experts in the area were also central to the findings. The results indicated a preference for visual supports and videos that were short and user friendly. This aligns with previous findings in the area, as visual resources are highlighted as being particularly useful for these populations (Rutherford et al., 2020). Visual resources can improve participation and communication, reduce anxiety and increase predictability. Predictability, routine and anxiety were particular concerns for the participants in this study.

Participants were also asked their opinion on the best way to disseminate resources developed. Trusted sources were the most highly regarded options. Schools, support groups and recommendations from resource users such as teachers or parents were the highest ranked options. Schools in particular are utilised by numerous organisations and initiatives to disseminate information and promote initiatives around education, wellbeing and extra-curricular activities. Online resources have been effectively disseminated through school settings in the past (Sam et al., 2020).

### Limitations

The current study demonstrates the utility of gaining expert consensus around challenges and resource needs for autistic children. While consensus was gained in just two rounds, it is important to note that attrition of participants from Round 1 to Round 2, particularly impacted on autistic participants. This means that their voices were not considered in the consensus gaining exercise which has implications for how we interpret our findings. Future research using Delphi Methods with similar participants should build in supports to reduce attrition. The Delphi method is useful in gaining consensus however it is possible that a different participant panel, in a different context or jurisdiction may have reached different conclusions. Future research could involve autictic children and young people in such studies. Furthermore, it is not to say that response options under the 70% cut off should be ignored, but that they may be placed at a lower priority. It should also be noted also that the Delphi method can be limited by the questions asked which in turn may be influenced by subconscious views or attitudes of the researchers. Efforts were made throughout the current study to involve a variety of different viewpoints and stakeholders in question development in order to minimise such biases.

### Impact and Implications

The continuous and drastic changes to everyday life throughout the pandemic have caused great disruption, and aiding this vulnerable population in the transition is of the utmost importance. The current findings gave insight into the resource needs to autistic children and young people as they, their families, educators and therapists navigate the COVID-19 pandemic in Ireland. Findings were used to develop resources that were made freely available during this time. While challenges and resource priorities were agreed by the expert panel, it is important to consider that resources to support similar challenges may look very different depending on the needs of autistic children with pre-verbal skills requiring different supports to those with moderate or very good verbal abilities. Such nuanced approaches must be taken to ensure that resources are useful. This is the first known study to assess need during the time of the COVID-19 pandemic through a Delphi method, ensuring that the needs of stakeholders are documented and can be addressed. Practically speaking, this facilitated us as researchers in producing resources to best serve the needs of the population at this time. As the pandemic continues to evolve, support needs to autistic populations will also. The current research demonstrates how consensus can be gained relatively simply and quickly in order to provide needs based direction on areas of priority.

## Conclusion

The onset of COVID-19 has brought with it a time of increased challenges for society. Autistic people are particularly vulnerable to the effects of the pandemic and associated restrictions. There is a significant need for targeted resources which these children and their families can avail of to navigate challenges which arise. With this in mind, the Delphi method provided a strong approach to ascertain the needs of autistic children and their families, and to achieve consensus on resources that could be utilised to ease transitions and other issues. Interventions targeting anxiety and changes to routine were most urgently needed based on the expert consensus in this study. Online dissemination is probably most effective due to the wide reach possible and its independence from pandemic restriction measures. A wide scale initiative across society is required in order to prevent our most vulnerable members from being affected disproportionately. However, that is not to say that other priorities identified in Round 1 but which did not achieve consensus in the top 3 ranking, should be ignored. Future research could make use of the Delphi method in order to rapidly assess the needs and target areas for autistic people, as well as other communities.

## Data Availability

Informed consent was not sought to share data in an open format due to the nature of the data collected. Support materials can be found at 10.6084/m9.figshare.17142455.v1

## Code Availability

N/A.
